# Association between serum uric acid and cardiovascular disease risk factors in adolescents in America: 2001-2018

**DOI:** 10.1371/journal.pone.0254590

**Published:** 2021-08-23

**Authors:** Qiqi Shi, Ran Wang, Huifeng Zhang, Yaping Shan, Ming Ye, Bing Jia

**Affiliations:** 1 Department of Pediatric Cardiothoracic Surgery, Children’s Hospital of Fudan University, Shanghai, China; 2 Department of Neonatology, Children’s Hospital of Fudan University, Shanghai, China; Holbaek Sygehus, DENMARK

## Abstract

SUA is associated with cardiovascular disease and cardiovascular disease risk factors in adults, including chronic kidney disease, coronary artery disease, stroke, diabetes mellitus, preeclampsia, and hypertension. A cross-sectional study was carried out among 11219 adolescents 12 to 18 years of age examined in the 2001–2018 National health and Nutrition Examination Survey. We examined the association between SUA and CVD risk factors. The overall mean SUA level was 5.00±1.24mg/dl. Restricted cubic spline analysis results revealed SUA was inversely associated with HDL-C and SPISE and positively associated with TC, TG, LDL-C, nonHDL-C, insulin, SBP and DBP after full adjustment. Multiple logistic analyses showed SUA level was independently associated with high TC, high TG, high nonHDL-C and low HDL-C (all p<0.05). Furthermore, females in the highest quartile of SUA had significantly higher odds for elevated BP (OR = 2.38, 95%CI:1.02–5.54, P<0.05) and high TC (OR = 2.22, 95%CI: 1.49–3.30, P<0.001), which not observed in males. Increased levels of SUA were associated with increased odds of various cardiovascular risk factors in American adolescents, especially females.

## Introduction

Cardiovascular disease (CVD) has been the leading cause of death all over the world [1]. Although manifest disease in childhood and adolescence is rare, risk factors and risk behaviors that accelerate the development of CVD begin in childhood, and it has been calculated that a reduction in cardiovascular risk factors in adolescence would lead to a reduction in the number affected by CVDs later in life [2]. Given the burden of CVD in both adolescents and adults, further elucidation of mechanisms and potential therapeutic targets is needed.

Although traditional risk factor-based prediction models have significant values for most of the CVD events, they do not account for the entirety of cardiovascular risks [3]. The presence of residual risk has necessitated the evaluation of new risk factors to improve CVD risk stratification.

Uric acid (UA) is the final product of dietary and endogenous purine metabolism [4]. Human are exposed to >50 times greater serum UA (SUA) concentrations than other mammals because of the lack of urate oxidase, an enzyme that is responsible for uric acid being converted into allantoin, which makes humans susceptible to hyperuricemia [5, 6]. Apart from being a diagnostic indicator of gout, increasing levels of SUA have been implicated in the pathophysiology of CVD risks in adults, such as hypertension, diabetes, metabolic syndrome, coronary artery disease and kidney disease [7, 8]. Epidemiologic studies have shown that the risk of cardiovascular disease and mortality related to elevated SUA is greater in adult females than in males [9–11]. Many research showed that sex-specific patterns of cardiac and vascular ageing begin early in life [12, 13]. However, few studies have examined whether SUA is related with CVD risks in teenagers of general population. Also, no large-scale studies have been performed to investigate the sex difference of the relationship between SUA and CVD risk factors in adolescents.

In the present study, we aimed to explore the associations between SUA and CVD risk factors in a large, nationally representative cohort of US adolescents using data from the National Health and Nutrition Examination Survey (NHANES), 2001–2018. An understanding of this relationship may prompt earlier surveillance of the SUA level and identify the populations at high risk of CVD.

## Materials and methods

### Study design

**Fig 1:**
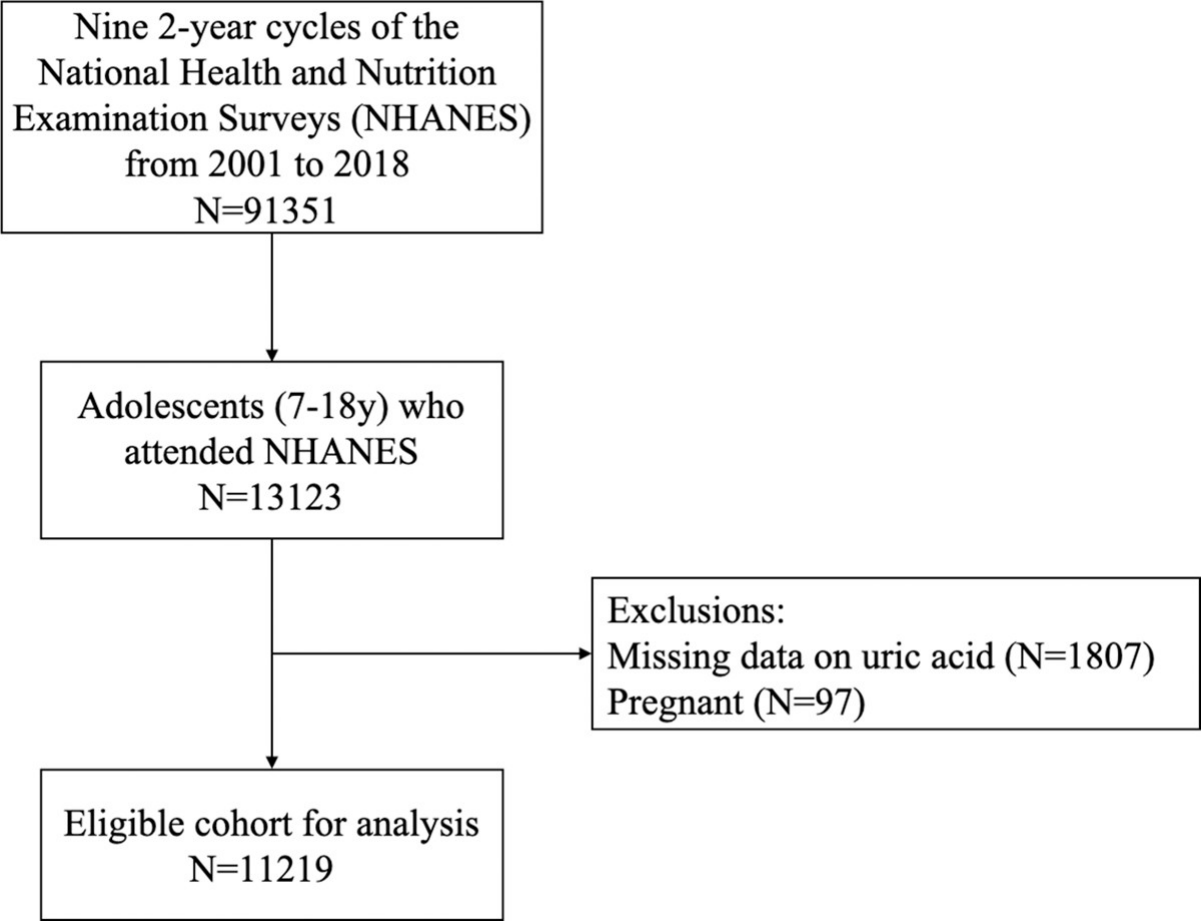
Eligible population and those included in the analyses of the relationship between serum uric acid and CVD risk factors in US adolescents.

The national center for health statistics, within the centers for disease control and prevention, conducted continuous NHANES 2-year cycles using a complex multistage sampling design to obtain a representative sample of the US civilian, non-institutionalized population aged ≥2 months. Details of the complex survey design are described on https://www.cdc.gov/nchs/nhanes/about_nhanes.htm and are briefly summarized herein. Adolescents aged 12 to 18 years who attended an examination during any NHANES cycle from 2001–2002 to 2017–2018 were included. This age range was chosen because SUA in the NHANES is examined beginning at age 12 years and older. This article is restricted to nonpregnant adolescent aged 12 to 18 years. We analyzed all available data, excluding individuals from particular analyses if relevant variables were missing. The process of data inclusion is presented in Fig 1.

NHANES study protocols were approved by the research ethics review board of the national center for health statistics. Written informed consent was obtained from the guardians of participants<18 years of age, and assent was obtained from those aged 12 to 17 years of age. The children’s hospital of Fudan University institutional review board waived the need for review because the research did not involve human participants. This study followed the reporting guidelines in the Strengthening the Reporting of Observational Studies in Epidemiology (STROBE) for cross-sectional study (S1 Checklist).

#### Demographics data and physical examination

Demographics data provided the participants’ age, gender, race/ethnicity and the poverty: income ratio (PIR). Race/ethnicity was categorized into 5 groups, Mexican American, other Hispanic, non-Hispanic white, non-Hispanic black and other. PIR is a representative of socioeconomic status and is expressed as the ratio of family income to the poverty threshold after correcting for inflation and family size. Screen time was asked by trained interviewers. The participants indicated the range of time the spent using computer (computer time) or watching TV (TV time) per day on average over the past 30 days.

Body mass index (BMI) was calculated as measured weight in kilograms divided by measured height in meters squared, and the age and sex standardized BMI (BMIZ) was calculated based on the Centers for Disease Control and Prevention BMI-for-age sex-specific growth charts [14]. Waist circumference (WC) was measured at the iliac crest to the nearest millimeter using a steel tape. Waist to height ratio (WHtR) was calculated as WC divided height.

#### SUA level evaluation

In NHANES, SUA was measured on a Roche Hitachi Model 917 or 704 Multichannel Analyzer in 2001 and a Beckman Coulter UniCel DxC 800&DxC 660i Synchron Clinical System in 2002–2018 using a colorimetric method. Serum creatinine level was measured with the same instruments using the Jaffe kinetic alkaline picrate method. Serum creatinine was corrected to standardize to a gold-standard reference method, as recommend by NHANES. The distribution of creatinine and uric acid results from the 2 laboratories were compared at the time transition, and no significant differences were observed.

### Evaluation cardiovascular disease risk factors

Cardiovascular risk factors were determined from serum samples and the physical examination. Total cholesterol (TC) was measured enzymatically in serum with a series of coupled reactions that hydrolyze cholesterol esters and oxidize the 3-OH group of cholesterol. Similarly, triglycerides (TG) and glucose were measured enzymatically in serum. High density lipoprotein cholesterol (HDL-C) was measured directly in serum with reagents from Roche/Boehringer-Mannheim Diagnostics. Serum low density lipoprotein cholesterol (LDL-C) level were estimated only on participants who fasted at least 8.5 hours in the morning session. LDL-C was calculated according to the Friedewald calculation: LDL-C = TC (mg/dl)—HDL-C (mg/dl)–triglycerides (mg/dl)/5. Non-HDL cholesterol (nonHDL-C) levels were calculated as TC (mg/dl) minus HDL-C (mg/dl). Serum creatinine level was measured using the Jaffe kinetic alkaline picrate method. Fasting plasma glucose (FPG) was measured by enzymatic method. Serum insulin was measured by a radioimmunoassay method. The Single Point Insulin Sensitivity Estimator (SPISE) was used as a surrogate measure of insulin sensitivity and calculated as 600*HDL-C^0.185^/ (TG^0.2^ ×BMI^1.338^) [15]. Estimated glomerular filtration rate (eGFR; milliliters per minute per 1.73 m^2^) was calculated via the creatinine-based formula of Schwartz: eGFR = k*(height in centimeters)/(serum creatinine in mg/dl), where k is 0.70 in boys and 0.55 in girls.

Blood pressure (BP) measurements were obtained by manual auscultation with a mercury-gravity manometer via a standardized protocol by trained physicians. Three BP readings were obtained after the participant had been seated, with feet on the ground and back supported, and resting quietly for 5 minutes. Each reading was obtained 30 seconds apart, and a fourth reading was obtained if 1 of the previous readings had been interrupted. Mean systolic (SBP) and diastolic blood pressures (DBP) for each participant were calculated from the recorded readings.

#### Definitions and diagnostic criteria

Adverse lipid concentrations were defined as follows: TC concentration of 200mg/dL or higher; HDL-C concentration lower than 40 mg/dL; TG and LDL-C concentration of 130mg/dl or higher; nonHDL-C concentration of 145 mg/dL or higher. Impaired fasting glucose (IFG) is defined when fasting plasma glucose (FPG) concentration between 100mg/dl to <126mg/dl. Diabetes is defined by either FPG>125mg/dl. In the present study, high FPG was defined as IFG or diabetes. According to Clinical Practice Guideline for Screening and Management of High Blood Pressure in Children and Adolescents published by American academy of pediatrics in 2017, hypertension was defined as average clinic measured SBP and/or DBP≥95th percentile (on the basis of age, sex, and height percentiles) on 3 consecutive visits. Because NHANES obtains BP measurements at a single study visit, and, thus, a formal diagnosis of hypertension is not possible. In this study, participants who were younger than 18 years old, were characterized as having “elevated BP” if the mean SBP and/or DBP percentile was≥95th percentile and “normal blood pressure” if the mean SBP and DBP percentiles were both <95th percentile [16]. Participants who were 18 years old, elevated BP was defined as BP≥ 140/90mmHg.

### Statistical analysis

Statistical analyses were performed using Stata Statistical Software, version 15 and R statistical software, version 3.5.3 (package ‘rms’). The normality of the data distribution was evaluated using Shapiro–Wilk tests. Data were expressed as mean±SD for continuous variables with a normal distribution, geometric mean (95% CIs) for skewed variables and were logarithmically transformed before analysis, and frequency (%) for categorical variables. To compare the differences in characteristics between two sexes, the χ2 test was applied for categorical variables, and Student’s t tests and rank sum tests were chosen for normally distributed and skewed variables, respectively. We computed SUA levels into quartiles according to its distribution in the present study. Considering the difference SUA levels between two genders, SUA levels were also classified into quartiles by sex, and a sex-specific analysis of SUA quartiles then performed. One-way ANOVA test and Kruskal-Wallis H test were chosen for normally distributed and skewed variables, respectively.

Spline regression was used to evaluate the association of SUA with cardiovascular risk factors with 3 knots located at the 10^th^, 50^th^, 90^th^ percentiles of SUA. Logistic regression was used to evaluate the association of SUA quartiles with cardiometabolic abnormalities. All of the regression analyses were adjusted for age, gender (not for sex-stratified analysis), race, PIR, daily TV and computer use time, BMIZ, WHtR and eGFR. The sensitivity analysis was conducted by model-based adjustments. We omitted those cases with the missing values of covariates. The statistical significance level was set at α = 0.05. All of the statistical analyses were 2 sided.

## Results and discussion

### 1. Characteristics of participants

Of eligible adolescents 12 to 18 years of age, 1807 were excluded for missing SUA, 97 were excluded because of pregnancy, resulting in a final sample size of 11219 (5767 males and 5452 females). Comparisons of the characteristics and laboratory profiles between boys and girls are shown in Table 1. The mean age of participants was 15.0 years old and there is no difference between boys and girls. BMI, WHtR, TC, LDL-C, HDL-C, nonHDL-C, fasting insulin and DBP in females were significantly higher than that in males while TG, fasting serum glucose, SPISE and SBP were significantly lower than that in boys. The overall mean of SUA level was 5.00±1.24mg/dl, which was significantly higher in males (5.58±1.21 mg/dl) than in females (4.38±0.94 mg/dl) (p<0.001).

**Table 1:** Comparison of characteristics of the study population between boys and girls in NHANES 2001–2018.

	N	Overall	Males	Females	P
Sample size (%)	11219	11219	5767(51.4%)	5452	
SUA (mg/dl)	11219	5.00±1.24	5.58±1.21	4.38±0.94	<0.001
Age (y)	11219	15.00±1.99	15.02±1.98	14.97±1.99	0.25
Race, N (%)					0.112
Mexican American	3044	3,044 (27.13)	1508 (26.15)	1,536 (28.17)	
Other Hispanic	850	850 (7.58)	425 (7.37)	425 (7.80)	
Non-Hispanic White	3151	3151 (28.09)	1649 (28.59)	1502 (27.55)	
Non-Hispanic Black	3146	3146 (28.04)	1645 (28.52)	1501 (27.53)	
Other race/multi-racial	1028	1028 (9.16)	540 (9.36)	488 (8.95)	
PIR, N (%)					0.069
<1	3109	3109 (27.71)	1555 (26.96)	1554 (28.50)	
> = 1	8110	8110 (71.5)	4212 (73.04)	3898 (71.50)	
TV time (h/d), N (%)					0.057
<1h	1232	1232 (15.28)	589 (14.39)	643 (16.19)	
1-3h	4917	4917 (60.97)	2537 (61.98)	2380 (59.93)	
> = 4h	1915	1915 (23.75)	967 (23.63)	948 (23.87)	
Computer time (h/d), N (%)					<0.001
<1h	3366	3366 (41.72)	1622 (39.59)	1744 (43.92)	
1-3h	3598	3598 (44.60)	1881(45.91)	1717 (43.24)	
> = 4h	1104	1104 (13.68)	594 (14.50)	510 (12.84)	
BMI (kg/m^2^)	11103	23.90±6.05	23.61±5.93	24.20±6.16	<0.001
BMIZ	11103	0.89±1.39	0.87±1.42	0.91±1.35	0.08
WC (cm)	10956	81.48±14.94	81.61±15.57	81.35±14.25	0.36
WHtR	10954	0.49±0.09	0.48±0.09	0.51±0.09	<0.001
HDL-C(mg/dl)	7553	52.28±12.46	50.53±12.30	54.14±12.36	<0.001
LDL-C (mg/dl)	4814	88.69±25.87	87.90±26.94	89.56±24.61	0.026
TC (mg/dl)	11211	158.21±29.57	156.18±30.02	160.36±28.93	<0.001
TG (mg/dl)	4911	82.15±58.67	84.32±67.64	79.76±46.77	0.007
nonHDL-C(mg/dl)	7553	104.26±28.88	103.62±29.80	104.93±27.86	0.049
Glu (mg/dl)	4960	93.91±17.47	95.80±19.13	91.82±15.17	<0.001
Insulin (uU/mL)	4909	14.44±12.66	13.72±12.41	15.23±12.88	<0.001
SPISE	3122	8.49±2.95	8.66±3.10	8.30±2.76	<0.001
SBP (mmHg)	9373	109.16±9.87	111.67±10.21	106.54±8.78	<0.001
DBP (mmHg)	9373	59.41±11.80	58.34±12.79	60.51±10.57	<0.001
eGFR (mL/min/1.73 m^2^)	9220	147.87±32.24	157.94±34.54	136.98±25.41	<0.001

SUA: serum uric acid; PIR: the poverty: income ratio; TV time: the average hours of watching TV per day over the past 30 days; Computer time: the average hours of a computer over the past 30 days; BMI: body mass index; BMIZ: body mass index Z score; WC: waist circumference; WHtR: waist to height ratio; HDL-C: high density lipoprotein cholesterol; LDL-C: low density lipoprotein cholesterol; TC: total cholesterol; TG: triglycerides; nonHDL-C: Non-HDL cholesterol; FPG: fasting plasma glucose; SPISE: Single Point Insulin Sensitivity Estimator; SBP: mean systolic blood pressures; DBP: mean diastolic blood pressures; eGFR: estimated glomerular filtration rate.

Data are expressed as the mean ± standard deviation (SD) or N and percent (%).

P-values are calculated by Student’s t-test for continuous variables and Chi-square test for categorical variables.

### 2. Associations between SUA levels and cardiometabolic parameters

Tables 2 and 3 present clinical characteristics and cardiovascular risk factors by quartiles of SUA levels in males and females, respectively. In male group, compared to subjects in the lowest quartile of SUA levels, those in the highest quartile had significantly older age (p<0.001), higher levels of BMIZ score (p<0.001), WHtR (p<0.001), LDL-C (p<0.001), non-HDL-C (p<0.001), TC (p<0.001), TG (p<0.001), fasting blood insulin (p<0.001), SBP (p<0.001) and DBP (p<0.001). Participants in male group with lower levels of SUA had higher HDL-C (p<0.001) and SPISE (p<0.001). In female group, similar results were found except for no significant difference were found in term of age between quartile groups (p = 0.813).

**Table 2:** Clinical characteristics and cardiometabolic risk factors by quartiles of serum uric acid in males.

	Overall	Q1	Q2	Q3	Q4	P-value
Sample size	5767	1571	1373	1436	1387	
SUA (mg/dl)	5.58±1.21	4.16±0.56	5.22±0.20^a^	5.93±0.23^b^^c^	7.17±0.78^d^^e^^f^	<0.001
Age (y)	15.02±1.98	14.08±1.97	15.05±1.91^a^	15.33±1.85^b^^c^	15.72±1.78^d^^e^^f^	<0.001
BMI (kg/m2)	23.61±5.93	20.65±4.06	22.56±4.88^a^	24.06±5.38^b^^c^	27.54±6.90^d^^e^^f^	<0.001
BMIZ	0.87±1.42	0.28±1.25	0.63±1.28^a^	0.96±1.32^b^^c^	1.66±1.45^d^^e^^f^	<0.001
Waist (cm)	81.61±15.57	73.48±10.73	78.70±12.58^a^	82.78±14.00^b^^c^	92.46±17.78^d^^e^^f^	<0.001
WHtR	0.48±0.09	0.45±0.06	0.46±0.07^a^	0.48±0.08^b^^c^	0.53±0.10^d^^e^^f^	<0.001
TC (mg/dl)	156.18±30.02	155.07±28.32	153.45±28.46	155.18±29.20	161.22±33.47^d^^e^^f^	<0.001
TG (mg/dl)	84.32±67.64	71.59±41.23	76.69±44.10	83.14±52.09^b^	109.61±110.27^d^^e^^f^	<0.001
LDL-C (mg/dl)	87.90±26.94	84.15±25.18	86.03±25.95	87.35±26.74	95.18±28.92^d^^e^^f^	<0.001
HDL-C (mg/dl)	50.53±12.30	55.30±12.96	51.29±11.53^a^	49.59±11.71^b^^c^	44.95±10.24^d^^e^^f^	<0.001
nonHDL-C (mg/dl)	103.62±29.80	98.13±26.73	100.64±27.60	103.56±29.81^b^	113.37±33.01^d^^e^^f^	<0.001
FBG (mg/dl)	95.80±19.13	95.49±21.00	94.54±10.74	96.34±26.04	96.93±13.93	0.139
Insulin (uU/mL)	13.72±12.41	11.19±9.59	12.22±10.25	13.27±11.10^b^	18.86±16.70^d^^e^^f^	<0.001
SPISE	8.66±3.10	10.45±2.99	9.09±2.63^a^	8.14±2.63^b^^c^	6.44±2.59^d^^e^^f^	<0.001
SBP (mmHg)	111.67±10.21	108.47±9.87	111.21±9.63^a^	112.55±9.98^b^^c^	114.69±10.32^d^^e^^f^	<0.001
DBP (mmHg)	58.34±12.79	56.91±13.25	58.20±12.23	58.42±13.10^b^	59.96±12.31^d^^e^^f^	<0.001

The abbreviations are the same as in Table 1

Data of CVD risk factors were expressed as mean ± standard deviation.

In males, Serum uric acid (mg/dl) quartiles were defined as follows: quartile 1 <4.9; quartile 2 4.9–5.5; quartile 3 5.6–6.3; quartile 4 >6.3.

P-values are calculated by One-way ANOVA test, pairwise comparison conducted with Bonferroni correction.

^a^ P<0.05 Q2 vs Q1

^b^ P<0.05 Q3 vs Q1

^c^ P<0.05 Q3 vs Q2

^d^ P<0.05 Q4 vs Q1

^e^ P<0.05 Q4 vs Q2

^f^ P<0.05 Q4 vs Q3.

**Table 3:** Clinical characteristics and cardiometabolic risk factors by quartiles of serum uric acid in females.

	Overall	Q1	Q2	Q3	Q4	P-value
Sample size	5452	1431	1442	1400	1197	
SUA (mg/dl)	4.38±0.94	3.30±0.37	4.06±0.17^a^	4.68±0.20^b^^c^	5.72±0.61^d^^e^^f^	<0.001
Age (y)	14.97±1.99	14.99±2.00	14.98±1.97	14.93±1.98	14.99±2.04	0.813
BMI (kg/m2)	24.20±6.16	21.97±4.50	23.14±4.99^a^	24.41±5.87^b^^c^	27.81±7.61^d^^e^^f^	<0.001
BMIZ	0.91±1.35	0.40±1.17	0.70±1.19^a^	0.99±1.28^b^^c^	1.66±1.45^d^^e^^f^	<0.001
Waist (cm)	81.35±14.25	76.13±10.44	78.87±11.84^a^	81.87±13.70^b^^c^	89.77±17.19^d^^e^^f^	<0.001
WHtR	0.51±0.09	0.48±0.06	0.49±0.07^a^	0.51±0.08^b^^c^	0.56±0.10^d^^e^^f^	<0.001
TC (mg/dl)	160.36±28.93	157.17±27.80	159.45±28.62	160.87±28.10^b^	164.64±30.99^d^^e^^f^	<0.001
TG (mg/dl)	79.76±46.77	69.52±36.22	78.51±50.66^a^	79.70±41.33^b^	92.44±54.90^d^^e^^f^	<0.001
LDL-C (mg/dl)	89.56±24.61	85.19±22.97	87.86±24.85	91.31±23.88^b^	94.19±25.96^de^	<0.001
HDL-C (mg/dl)	54.14±12.36	57.37±12.42	55.23±12.52^a^	53.35±11.59^b^^c^	49.89±11.63^d^^e^^f^	<0.001
nonHDL-C (mg/dl)	104.93±27.86	99.77±26.25	101.91±26.62	106.80±26.94^b^^c^	112.58±30.32^d^^e^^f^	<0.001
FBG (mg/dl)	91.82±15.17	92.06±21.53	91.64±15.66	90.95±9.14	92.87±11.74	0.182
Insulin (uU/mL)	15.23±12.88	12.90±10.24	13.87±11.11	15.40±12.49^b^	19.18±16.52^d^^e^^f^	<0.001
SPISE	8.30±2.76	9.49±2.60	8.68±2.43^a^	8.03±2.59^b^^c^	6.91±2.88^d^^e^^f^	<0.001
SBP (mmHg)	106.54±8.78	105.54±8.27	105.84±8.90	106.89±8.73^b^^c^	108.13±9.04^d^^e^^f^	<0.001
DBP (mmHg)	60.51±10.57	59.89±10.26	60.33±10.78	60.54±10.77	61.43±10.38^de^	0.006

The abbreviations are the same as in Table 1

Data of CVD risk factors were expressed as mean ± standard deviation.

In females, Serum uric acid (mg/dl) quartiles were defined as follows: quartile 1<3.8; quartile 2 3.8–4.3; quartile 3 4.4–5.0; quartile 4 >5.0.

P-values are calculated by One-way ANOVA test, pairwise comparison conducted with Bonferroni correction.

^a^ P<0.05 Q2 vs Q1

^b^ P<0.05 Q3 vs Q1

^c^ P<0.05 Q3 vs Q2

^d^ P<0.05 Q4 vs Q1

^e^ P<0.05 Q4 vs Q2

^f^ P<0.05 Q4 vs Q3.

### 3. Associations of SUA and cardiovascular risk factors

**Fig 2:**
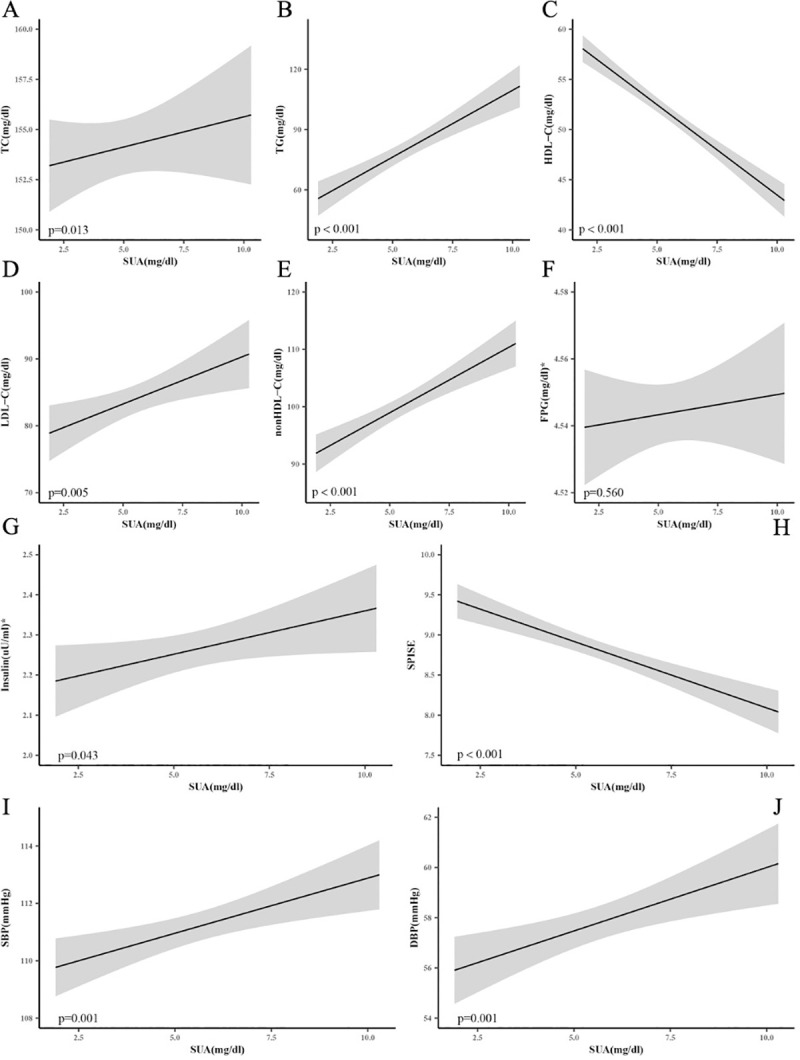
Plots show the non-linear regression models for SUA and cardiovascular risk in US adolescents 2001–2018. Analyses were adjusted for age, gender, race, PIR, TV time, computer time, BMIZ, WHtR and eGFR. SUA, serum uric acid; TC, total cholesterol; TG, triglycerides; HDL-C, high density lipoprotein cholesterol; LDL-C, low density lipoprotein cholesterol; nonHDL-C, Non-HDL cholesterol; FPG, fasting plasma glucose; SPISE, Single Point Insulin Sensitivity Estimator; SBP, mean systolic blood pressures; DBP, mean diastolic blood pressures. *Data were logarithmically transformed.

Non-linear regression models for the associations of SUA with metabolic risk factors in US adolescents were shown in Fig 2. There was an inverse association between SUA and both HDL-C and SPISE after adjusting for age, gender, race, PIR, daily computer and TV use time, BMIZ, WHtR and eGFR. SUA was positively related with TC, TG, LDL-C, nonHDL-C, insulin, SBP and DBP after full adjustment. However, there was no association between SUA and FPG.

### 4. Associations between SUA status and cardiometabolic abnormalities

Prevalence of adverse concentrations of TC, TG, LDL-C, HDL-C, and nonHDL-C were 8.36% (95%CI, 7.86–8.88), 11.83% (95%CI, 10.96–12.76), 1.72% (95%CI, 1.39–2.13), 13.84% (95%CI, 13.07–14.63) and 8.94% (95%CI, 8.31–9.60), respectively; prevalence of elevated BP was 2.7% (95%CI, 2.4–3.0); prevalence of high fasting glucose was 18.69% (95%CI, 17.63–19.80).

**Fig 3:**
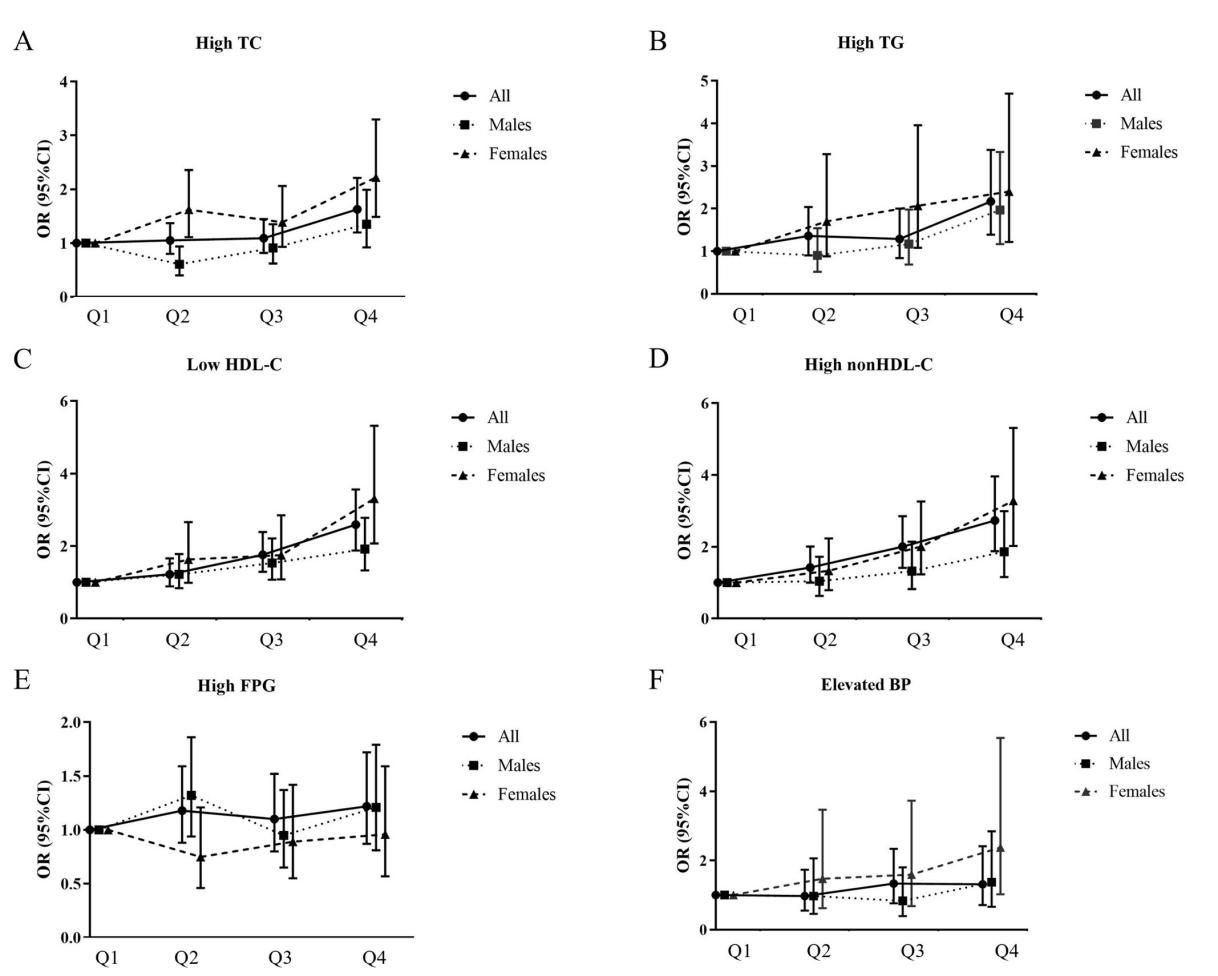
Adjusted OR and 95%CI for quartiles SUA and metabolic abnormalities in US adolescents 2001–2018, stratified by sex. Analyses were adjusted for age, gender (not for adjusted in stratified analysis), race, PIR, TV time, computer time, BMIZ, WHtR and eGFR. In all, serum uric acid (mg/dl) quartiles were defined as follows: quartile 1 <4.2; quartile 2 4.2–4.9; quartile 3 5.0–5.8; quartile 4 >5.8. In males, serum uric acid (mg/dl) quartiles were defined as follows: quartile 1 <4.9; quartile 2 4.9–5.5; quartile 3 5.6–6.3; quartile 4 >6.3. In females, serum uric acid (mg/dl) quartiles were defined as follows: quartile 1 <3.8; quartile 2 3.8–4.3; quartile 3 4.4–5.0; quartile 4 >5.0. Reference, quartile 1.

ORs of cardiometabolic abnormalities across different SUA status groups were examined using a multiple logistic regression analysis. After multivariate adjustment, compared with the lowest uric acid quartile, a significantly higher odds of high TC, high TG, high nonHDL-C and low HDL-C was noticed in participants in the highest quartile of SUA levels. In sensitivity analysis, we conducted logistic regression by adjusting different covariates. The associations between SUA and cardiovascular risk factors remained almost unchanged (S1 Table). As SUA levels in males were significantly higher than in females, we stratified the population by gender. As shown in Table 2, quartiles of SUA in males were 4.16±0.56mg/dl, 5.22±0.20 mg/dl, 5.93±0.23 mg/dl and 7.17±0.78 mg/dl, respectively. While quartiles of SUA in girls were 3.30±0.37mg/dl, 4.06±0.17 mg/dl, 4.68±0.20 mg/dl and 5.72±0.61 mg/dl, respectively. Furthermore, the logistic regression analyses were stratified by gender. As shown in Fig 3, in both males and females, the highest quartile was significantly associated with the presence of high TG, low HDL-C and high nonHDL-C. However, compared with the lowest SUA quartile, only females in the highest quartile had a higher OR of elevated BP and high TC, the association did not exist in males.

## Discussion

This is a study in a large, representative, multi-ethnic, population-based sample of adolescents to examine the association of SUA with cardio-metabolic risk biomarkers. Independent of traditional potential confounders, SUA was detrimentally associated with several cardiometabolic parameters, including TC, TG, LDL-C, HDL-C, nonHDL-C, SPISE, SBP and DBP. Furthermore, elevated SUA levels in US adolescents were independently associated with increased risks of abnormal concentrations of TC, TG, HDL-C, nonHDL-C and elevated BP after adjustment for other potential confounding risk factors. This finding provides a pivotal evidence that elevated SUA may increase CVD risk in adolescents. Interestingly, we also found that elevated SUA levels only increased risks of elevated BP and abnormal concentration of TC in female adolescents rather than males.

Many studies have evaluated the relationship between SUA and BP in both adults and children and indicated that hyperuricemia is associated with hypertension [17, 18]. Similar to the previous studies, our results have proved that SUA concentrations were positively associated with SBP and DBP after adjusting the age, gender, race, PIR BMIZ and WHtR. Furthermore, elevated SUA level was found to be correlated with elevated BP in female adolescents while no similar correlation was found in males after full adjustment. This finding is not quite consistent with the previous NHANES study which proved that elevated SUA levels were associated with elevated BP in both males and females [19]. This contradiction may be explained by different stratification ranges adopted. Some experimental studies have revealed the mechanisms of the association between SUA and BP. As reviewed by Gjin Ndrepepa [8], UA inhibits nitric oxide availability, which plays a crucial role in the regulation of BP. In addition, SUA can also promote vascular smooth cell proliferation and increase the expression of angiotensin II in vascular endothelial cells.

In the present study, we found that SUA was inversely related with SPISE, which indicates the insulin sensitivity, after adjusting different cofounders. The result was in consistence with the previous clinical studies in adults. The precise mechanisms have not been fully elucidated. Experimental studies have provided evidence that UA may impair insulin signaling in the liver, skeletal muscles and adipose tissues, thus contributing to the pathogenesis of hepatic and systematic insulin resistance-related disorders such as systemic inflammation, liver steatosis, and, eventually, type 2 diabetes [20–23].

Dyslipidemia is an important risk factor of CVD. Results of our study found that higher level of SUA might be associated with dyslipidemia (high TC, TG and nonHDL-C and low HDL-C). Further analysis revealed that odds radio in female adolescents were higher than males although the later had a higher level of SUA. Consistent with our results, many studies revealed that SUA was a better predictive factor of CVD and CVD risks among women, but not men [24–26].

We are interested in the sex-based differences in SUA levels and the association with metabolic abnormalities. There are data indicating that sex hormones may play a critical role in insulin sensitivity and body fat distribution, which may partly explain the sex difference of SUA levels. As reported before, estrogens have uricosuric effect on kidney to promote the excretion of UA and regulate the metabolism of lipid. However, our participants, who are going through puberty, may not have the balance of sex hormones characteristic of adults. In our study, female adolescents have a higher BMIZ and WHtR than males, which indicates that abdominal obesity might be more frequent in the former group. As we all known, abdominal obesity has already been proved to be a critical risk factor of metabolic diseases. This may partly explain the gender differences between SUA and CVD risks. However, the mechanism for this phenomenon is not clearly and completely understood. Further studies elucidating the exact mechanism of gender specific association of SUA and metabolic abnormality are still needed.

Our study has some strengths. First, this study contains a very large sample of US youth recruited from NHANES which applied rigorous quality controls to the procedures. Second, we adjusted for most potential confounders and effect modifiers. Third, we handled the target independent variable as both a continuous variable and as a categorical variable. Such an approach can reduce the contingency in the data analysis and enhance the robustness of results.

There are some limitations to our study. First, the main limitations of this is that data are cross-sectional, and no intervention can be applied to infer the causal associations. Second, the definition of elevated blood pressure is based on a single physical examination visit and does not necessarily signify that the participant has hypertension. However, elevated blood pressure may be indicative of a prehypertensive state, and it is known that prehypertension is a risk factor for the development of hypertension in children and other cardiovascular outcomes in adulthood. Third, some other confounders such as smoking status and physical activity were not included in or controlled for in our analyses. Fourth, pubertal stage was not evaluated because relevant data was not available.

## Conclusions

In summary, increased levels of SUA were associated with increased odds of various cardiovascular risk factors in American adolescents, especially females. It may be useful for female adolescents with higher SUA to pay more attention to prevent CVD.

## Supporting information

S1 Checklist STROBE statement—checklist of items that should be included in reports of *cross-sectional studies*. (DOC)

S1 Table Multivariable associations of SUA with cardiovascular risk factors in U.S. adolescents. Model 1: adjusted for age, gender, race and PIR. Model 2: model 1 plus adjusted for BMIZ and WHtR. Model 3: model 2 plus adjusted for TV time, computer time and eGFR.
(XLSX)
